# Radiotherapy may exacerbated anti‐programmed cell death 1 treatment induced vitiligo: A case report

**DOI:** 10.1002/ski2.287

**Published:** 2023-09-26

**Authors:** Chengqian Chen, Qihang Chang, Bo Wang, Yaqi Wang, Zhen Zhang, Xiuli Wang

**Affiliations:** ^1^ Institute of Photomedicine Shanghai Skin Disease Hospital School of Medicine Tongji University Shanghai China; ^2^ Department of Dermatology University of Michigan Ann Arbor Michigan USA; ^3^ Department of Radiation Oncology Fudan University Shanghai Cancer Center Shanghai China; ^4^ Department of Oncology Shanghai Medical College Fudan University Shanghai China; ^5^ Shanghai Clinical Research Center for Radiation Oncology Shanghai China; ^6^ Shanghai Key Laboratory of Radiation Oncology Shanghai China

## Abstract

Immunotherapy with programmed cell death 1 (PD‐1) checkpoint inhibitors combined with chemoradiotherapy shows great potential for cancer treatment and is getting extensively researched. However, a plethora of immune‐related adverse events (irAEs) has been observed during anti‐PD‐1 treatment, including cutaneous adverse events, such as vitiligo and pruritus. These adverse events may lead to treatment discontinuation. When anti‐PD‐1 treatment is combined with radiotherapy (RT), irAEs may be exacerbated. Here we present a case report of an elderly patient with stage IIIb rectal cancer, who developed PD‐1 inhibitor‐associated vitiligo. After a session of RT, vitiligo lesions enlarged shortly thereafter. After discontinuation of anti‐PD‐1 treatment, vitiligo lesions and pruritus quickly improved with appropriate treatment. The rectal cancer achieved clinical complete response with no sign of recurrence or metastasis during follow‐up. Considering the similar mechanism of anti‐PD‐1 treatment in targeting cancer and in inducing irAEs, cutaneous adverse events may be associated with favourable clinical response. Additionally, cutaneous irAEs aggravated by RT in this patient may suggested significant immune activation, which may occasionally contribute to tumour regression and favourable clinical outcome.

## Graphical abstract

Immunotherapy often leads to cutaneous immune‐related adverse events. When combined with radiotherapy, programmed cell death 1 inhibitor‐associated vitiligo and pruritus were exacerbated, which suggests significant immune activation and may be associated with favourable antitumour response. Besides, this case provides an idea for the diagnosis and treatment of refractory vitiligo caused by immunotherapy.

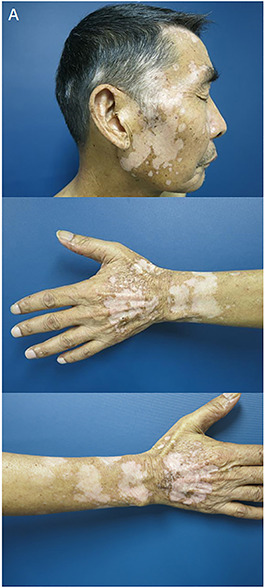

## 1 INTRODUCTION

The management of malignancies with immune checkpoint inhibitors (ICI) is gaining remarkable interest all over the world. Programmed cell death 1 (PD‐1) inhibitors work by blocking the PD‐1 immune checkpoint pathway to reactivate T cell‐mediated antitumour immunity. With reactivation of cellular immunity, numerous autoimmune‐like adverse events have been reported. Pruritus is one of the most prevalent immune‐related adverse events (irAEs); other common cutaneous adverse events include vitiligo, morbilliform, psoriasiform and lichenoid eruptions. In a comprehensive systematic review and meta‐analysis of 125 clinical trials, comprising a total of 20 128 patients, a significant incidence of vitiligo was reported, with rates reaching as high as 3.26% (95% CI, 2.80%–3.79%).^1^, ^2^ These adverse events may delay the subsequent dosing of treatment, disrupting treatment schedule and efficacy. When adverse events are severe, treatment will be discontinued, and hospitalization is often required.

PD‐1 blockade has proven to be highly effective for microsatellite instability‐high or mismatch repair deficient colorectal cancer.^3^ However, patients with microsatellite stable colorectal cancer account for the majority, who may not benefit from immunotherapy alone. Neoadjuvant chemoradiotherapy combined with total mesorectal excision, the traditional therapy for rectal cancer, has been challenged in recent years by the growing demand for efficacy and anus preservation. The patients who achieved clinical complete response after neoadjuvant therapy can choose the non‐operative ‘watch and wait’ strategy, so that significantly improving their quality of life. In such context, PD‐1 inhibitors combined with neoadjuvant chemoradiotherapy are widely studied, which showed that the efficacy of PD‐1 inhibitors is enhanced by local radiotherapy (RT) through multiple mechanisms. Meanwhile, potential or existing irAEs associated with anti‐PD‐1 therapy may develop or exacerbate.

Here we present an elderly patient who developed vitiligo after receiving toripalimab (PD‐1 inhibitor) treatment; vitiligo lesions were aggravated and accompanied by the pruritus after RT.

## 2 CASE REPORTS

A patient in his 60s with stage IIIb rectal cancer received combination therapy (chemotherapy + PD‐1 inhibitor: Oxaliplatin 200 mg d1 + capecitabine 1000 mg/m^2^ bid d1–d14 + toripalimab 240 mg d1) every 3 weeks. Two weeks after first treatment, scattered depigmented macules started to appear near hairline. One month after the second cycle of combination therapy, the patient underwent a short‐course RT (2500 cGy/5 Fx) prior to routine treatment. The depigmentation rapidly enlarged shortly after RT, spread to head, chest, back and extremities and was accompanied by intense itch. Depigmented rash continued to enlarge with each cycle of RT, reaching its worst condition about a week after their last RT session (total body surface area ∼10%).

**FIGURE 1:**
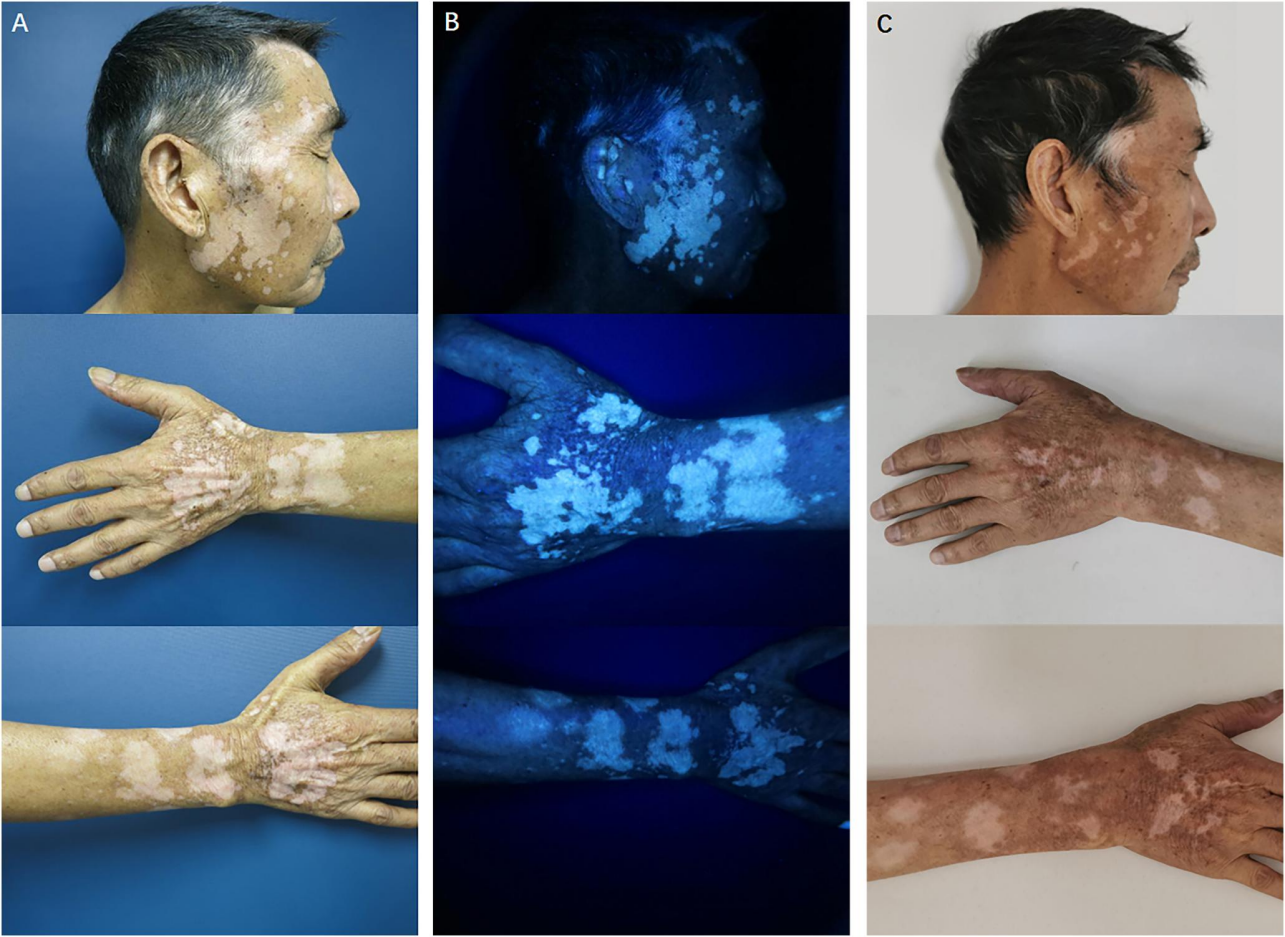
Clinical presentation before treatment (a, b) and after 1‐month follow‐up (c). (a) Well‐defined coalescing hypopigmented macules and patches on face, ear, scalp, dorsal hand and dorsal forearm, with leukotrichia at skin lesions on the head and face before treatment. (b) Lesions under Wood's lamp before treatment. (c) Repigmentation of the lesions after 1‐month follow‐up.

The fourth cycle of therapy was suspended due to acute kidney injury (AKI), for which the patient was hospitalized and received 1 week course of methylprednisolone. During course of systemic steroid, their pruritus showed improvement while widespread depigmentation remained. Three weeks later, a cycle of chemotherapy (oxaliplatin + capecitabine) was resumed without PD‐1 inhibitors. The following week, this patient presented to dermatology clinic with improved patches of vitiligo and no pruritus. Based on patient's history of anti‐PD‐1 therapy, as well as rash morphology and Wood's lamp examination (Figure 1a,b), the diagnosis of cutaneous irAEs and vitiligo were made. Patient received daily topical halometasone cream and enliutasol film coating (contains salicylic acid, clobetasol propionate and dithranol) for treatment. One month after above treatment, remarkable repigmentation was observed (Figure 1c). As of the time of this manuscript (3 months post treatment), his rectal cancer has achieved continuous clinical complete response with no sign of recurrence or metastasis, and remains on active surveillance (Table 1).

**TABLE 1:** Timeline of events and treatment for the patient.

2022/1/7	The patient presented to the outpatient clinic with a 1‐month history of haematochezia
2022/1/13	Colonoscopy showed a bulging lesion 5–8 cm from the anus, pathology: Adenocarcinoma
2022/2/16	Rectal MR: Rectal MT about 7.0 cm from the anus. T3aN1a, MRF‐, EMVI‐
2022/2/23	First cycle of combination therapy: XELOX + PD‐1
2022/3/7	Scattered depigmented macules began to appear near hairline
2022/3/22	Second cycle of combination therapy: XELOX + PD‐1
2022/4/18	Short course of radiotherapy for the primary rectal lesion + pelvic lymphatic drainage area was started (2500 cGy/5 Fx)
2022/4/19	The depigmentation rapidly enlarged, spread to head, chest, back and extremities and was accompanied by intense itch
2022/4/30	Depigmented rash continued to enlarge with each cycle of RT, reaching its worst condition about a week after their last RT session (TBSA ∼10%)
2022/5/9	Third cycle of combination therapy: XELOX + PD‐1
2022/5/30	Rectal MR: tumour is smaller than before. T2N0, MRF‐, EMVI‐
2022/6/10	CRE: 178 μmol/L, PROBNP: 111.00 pmol/L, FT4: 6.37 pmol/L, TSH: >100.0000 mIU/L, TGAb: 23.77 IU/mL, TPOAb: 67.46 IU/mL. The patient received methylprednisolone, levothyroxine sodium tablets and symptomatic therapy
2022/6/16	Pruritus improved while widespread depigmentation remained (TBSA ∼10%)
2022/7/11	Fourth cycle of therapy: XELOX (without PD‐1)
2022/7/18	The diagnosis of cutaneous irAEs and vitiligo was made. Patient received daily topical halometasone cream and enliutasol film coating (contains salicylic acid, clobetasol propionate and dithranol) for treatment
2022/8/1	Fifth cycle of therapy: XELOX (without PD‐1)
2022/8/22	No newly appearing depigmented spots, and remarkable repigmentation was observed (TBSA ∼5%)
2022/8/23	Anal finger examination: No obvious masses were detected
2022/8/24	Colonoscopy: About 6 cm from the anus see post‐radiotherapy scars, thickened vascular texture, no obvious ulcers or neoplastic organisms. Examination impression: Rectal cancer after neoadjuvant radiotherapy, cCR possible
2022/8/24	Rectal MR: lesion currently unremarkable
2022/8/26	PET‐CT: No significant foci of abnormally elevated FDG metabolism in the rectum; no significant abnormally elevated FDG metabolism
2022/11/27	Persistent cCR with no signs of recurrent metastasis

Abbreviations: cCR, complete clinical response; FDG, fluorodeoxyglucose; irAEs, immune‐related adverse events; PD‐1, programmed cell death 1; PET‐CT, positron emission tomography‐computed tomography; RT, radiotherapy; TBSA, total body surface area; TSH, thyroid‐stimulating hormone.

## 3 DISCUSSION

Anti‐PD‐1 treatment‐induced vitiligo is potentially associated with the cross‐reaction against antigens shared by cancer cells (especially in melanoma) and normal melanocytes, such as MART‐1, GP100, TRP1‐2 or tyrosinase.^1^ While vitiligo vulgaris is eventually completely devoid of epidermal melanocytes, pigmentation of terminal hairs is usually preserved. This observation suggests existence of an intact hair bulb, and an intact bulge melanocyte stem cell reservoir in associated hair follicles, both of which are spared from the effects of the immune attack and critical for hair pigmentation.^4^, ^5^ Hair follicle immune privilege can sequester highly immunogenic autoantigens from immune recognition, such as melanogenesis‐related self‐peptides; one of the main potential mechanisms is that PD‐L1 expressed in hair follicles delivers an inhibitory signal through PD‐1 present on T cells to induce cell anergy.^6^ Due to above mechanisms, PD‐1 blocking antibody treatment for cancer often causes vitiligo with leukotrichia,^7^ and also observed in our case (Figure 1).

Besides directly inducing cancer cell death, RT could activate anti‐tumour immunity not only at the site of treatment but also systematically to inhibit both primary and secondary tumours; the latter is named ‘abscopal effect’, which has been shown to be mediated by multiple immune mechanisms.^8^, ^9^ Theoretically, RT can induce a stronger abscopal effect when in combination with ICI, and the systemic efficacy of ICI can be enhanced by abscopal effect similarly. Hence, RT and ICI have synergistic effect, and that was proved by preclinical and clinical studies.^8^, ^9^ Cutaneous adverse events usually positively associate with better clinical response.^10^, ^11^, ^12^ In several studies, combination of ICI and RT resulted in increased incidence and severity of irAE‐like systemic adverse effects (such as pneumonitis),^9^, ^12^, ^13^ suggesting that the mechanism by which local RT exacerbates systemic irAEs may also be explained by abscopal effect. In addition, given that AKI is also a common complication in patients receiving ICI treatment as well as recovery of creatinine level after systemic steroid, AKI in this case after RT may also be attributed to ICI.

Several cases have been reported of patients who developed vitiligo on RT‐treated field. The suggested mechanism is radiation‐induced free radical‐mediated damage and subsequent apoptosis of susceptible melanocytes.^14^ Melanocyte death induced by RT exposes autoantigens to the host immune system, which may initiate systemic anti‐melanocyte responses.^15^ Consistent with the fact that abscopal tumour regression is rare, patients with extensive vitiligo are rarely seen after local RT. However, that may be complicated by ICI‐induced systemic reactivation of T cell.

In summary, both anti‐PD‐1 therapy and RT have the capacity to stimulate the immune system. When used in combination, they may exhibit a synergistic effect, which could lead to better efficacy, but more severe adverse events. Cutaneous irAEs aggravated by RT in the reported patient may suggested significant immune activation, which could be consistent with persistent tumour regression. However, this is only an observation in one patient and more studies are needed to confirm this finding.

## CONFLICT OF INTEREST STATEMENT

None to declare.

## AUTHOR CONTRIBUTIONS

**Chengqian Chen**: Data curation (equal); writing – original draft (equal). **Qihang Chang**: Writing – original draft (equal). **Bo Wang**: Writing – review & editing (lead). **Yaqi Wang**: Writing – review & editing (supporting). **Zhen Zhang**: Project administration (equal). **Xiuli Wang**: Project administration (equal).

## ETHICS STATEMENT

Not applicable.

## Data Availability

Data sharing is not applicable to this article as no new data were created or analyzed in this study.
